# 

**Published:** 1897-02-27

**Authors:** 


### EARLY EDITION.

**Notices of Births, Deaths, and Marriages** are inserted in this column at a charge of 2s. 6d. for an announcement not exceeding four lines.

**Death.**

MAGGIE MILNE.—On February 13th, of pneumonia, contracted in the discharge of her duties at the New Infirmary, Walsall. For two years nurse at the Home for Incurables, Liverpool, and for the last seven months The Infirmary, Walsall. (1,253)

### NOTICE TO CORRESPONDENTS.

All MS., Letters, Books for Review, and other matters intended for the Editor should be addressed The Editor, "The Hospital," The Hospital Building, 28 & 29, Southampton Street, Strand, W.C.

The Editor cannot undertake to return rejected MS., even when accompanied by stamped directed envelope.

**Notice to Advertisers and Subscribers.**

All Advertisements, Orders for Copies of The Hospital, and Business Communications should be addressed to **THE MANAGER, (Not to The Editor),** The Hospital, The Hospital Building, 28 & 29, Southampton Street, Strand, London, W.C.

The Cost of Subscription, post free, is as follows:—Per week, 2½d.; per quarter, 2s. 8d.; per half-year, 5s. 3d.; per annum, 10s. 6d. Foreign:—Per annum, 15s. 2d.; payable, in all cases, in advance.

**NOTICE.—Vol. XX. of "The Hospital" (April to September, 1896), in handsome cloth binding, is now on sale at the Office of this Journal, price 7s. 6d. Cases for binding the Numbers contained in this Volume, 1s. 6d. Index to Vol. XX., 2½d., post free.**

**The Monthly Part for March will be ready on March 1st, Price 6d., by post 8d.**

### BOOKS RECEIVED.

THE F. A. DAVIS COMPANY.
"Autoscopy of the Larynx and Trachea." By Alfred Kirstein, M.D.

E. MARLBOROUGH AND CO.
"Queen Victoria's Bounty: A Suggestion to John Bull."

CHATTO AND WINDUS.
"Herbert Fry's Royal Guide to London Charities."

**Periodicals Received.**—Humanitarian, London, Literary Digest, Guy's Hospital Gazette, Appleton's Popular Science Monthly, Knowledge.

### THE STUDENT OF MEDICINE.

### APPOINTMENTS.

E. W. Adams, F.R.C.S.Eng., L R.C.P.Lond., appointed Medical Officer of Health to the Slough Urban District Council. N. E. Aldridge, M.B., C.M.Edin., D.P.H.R.C.P. & S.Eng., appointed Physician to the Royal South Hants Infirmary, Southampton. H. L. Barnard, M.B., M.S.Lond., F.R.C.S.Eng., appointed Surgical Registrar of the London Hospital. H. A. Collinson, M.B., B S., appointed Surgeon to the Richmond Rolling Mills Limited, Stockton-on-Tees. G. M. Edmond, appointed Physician to the Aberdeen Royal Infirmary. W. H. Fawcett, L.R.C.P., L.R.C.S.Edin., D.P.H.R.C.S.Eng., appointed Medical Officer for the Wimborne District of the Wimborne and Cranborne Union.

### HER MAJESTY'S ARMY AND INDIAN MEDICAL SERVICES.

The Director-General of the Army Medical Department asks us to publish the following list of successful candidates for commissions in the Army Medical Staff at the recent examination in London: S. L. Cummins, 2,272 marks; C. H. Hopkins, 2,207; J. McArdle, 2,159; P. Mackessack, 2,158; L. J. C. Hearn, 2,143; J. McD. McCarthy, 2,065; E. Brodribb, 2,055; J. G. Walker, 2,005; H. G. F. Stallard, 1,948; Q. D. Jephson, 1,887; H. L. W. Norrington, 1876; J. Poe, 1,847; J. Crean, 1,828; A. W. N. Bowen, 1,809.

